# Rapid whole genome sequencing of Miyazaki-Bali/2007 *Pteropine orthoreovirus* by modified rolling circular amplification with adaptor ligation – next generation sequencing

**DOI:** 10.1038/srep16517

**Published:** 2015-11-12

**Authors:** Harpal Singh, Tomoki Yoshikawa, Takeshi Kobayashi, Shuetsu Fukushi, Hideki Tani, Satoshi Taniguchi, Aiko Fukuma, Ming Yang, Masami Sugamata, Masayuki Shimojima, Masayuki Saijo

**Affiliations:** 1 Department of Intelligent Mechanical Systems, Graduate School of Systems Design, Tokyo Metropolitan University, Tokyo, 192-0065, Japan; 2 Special Pathogens Laboratory, Department of Virology 1, National Institute of Infectious Diseases, Tokyo, 208-0011, Japan; 3 Laboratory of Viral Replication, International Research Center for Infectious Diseases, Research Institute for Microbial Diseases, Osaka University, Osaka, 565-0871, Japan; 4 Department of Hygiene and Public Health, Graduate School of Human Health Sciences, Tokyo Metropolitan University, Tokyo, 192-0397, Japan

## Abstract

The emergence of orthoreoviruses as the causative agent of human respiratory illness over the past few years has led to a demand to determine their viral genome sequences. The whole genome sequencing of such RNA viruses using traditional methods, such as Sanger dideoxy sequencing following rapid amplification of cDNA ends presents a laborious challenge due to the numerous preparatory steps required before sequencing can commence. We developed a practical, time-efficient novel combination method capable of reducing the total time required from months to less than a week in the determination of whole genome sequence of *Pteropine orthoreoviruses* (PRV); through a combination of viral RNA purification and enrichment, adaptor ligation, reverse transcription, cDNA circularization and amplification, and next generation sequencing. We propose to call the method “modified rolling circular amplification with adaptor ligation – next generation sequencing (mRCA-NGS)”. Here, we describe the technological focus and advantage of mRCA-NGS and its expansive application, exemplified through the phylogenetic understanding of the Miyazaki-Bali/2007 PRV.

Viral genome sequence determination is crucial in the taxonomy of viruses, the understanding of viral origins and viral evolutionary history^1^. Generally, DNA sequencing is based on the application of first generation sequencing methods such as the Sanger dideoxy technique^2^,^3^. This is, however, laborious for several families of virus, such as orthoreovirus and influenza viruses, whose genomes are segmented into 6–12 parts^4^,^5^,^6^. The difficulty in obtaining full-length sequences, especially those of the terminal ends of both segmented and non-segmented viruses, necessitates the use of other methods such as rapid amplification of cDNA ends (RACE). While RACE addresses these constraints, the method must be carried out individually for each of the genomic segments which can be more burdensome for segmented viruses^7^. In the field of virology, recent advances in nucleotide sequence determination by next-generation sequencing (NGS) have allowed for the rapid determination of whole genome sequences of viruses. Notably, this advancement has facilitated greater understanding of previously unknown aspects of viral evolution and dynamics^8^. There are, however, limitations to the use of NGS, such as the prerequisite of obtaining viral genomes of high quality and purity, without which, a thorough and unbiased understanding of viral evolution and dynamism would be affected^8^,^9^, which this study aimed to address.

A combination method for genome sequencing, “modified rolling circular amplification with adaptor ligation – next generation sequencing” (mRCA-NGS), was developed and applied in the determination of the whole genome sequence characterization of an emerging strain of *Pteropine orthoreoviruses* (PRV), Miyazaki-Bali/2007^10^. The 10 discrete segments of the PRV genome consist of 3 large (L) (L1-3), 3 medium (M) (M1-3), and 4 small (S) (S1-4) size-class RNA segments encoding 12 proteins^6^,^11^. There has been an increasing number of reports over the past few years of patients with acute respiratory tract infections caused by PRV^6^,^10^,^12^,^13^,^14^,^15^,^16^, which are known to be phylogenetically related to previously isolated bat-borne orthoreoviruses^4^,^5^,^6^,^17^,^18^,^19^,^20^,^21^,^22^, thus making the genetic divergence of these strains of interest.

This method overcomes the challenge of obtaining high quality, high purity, and complete coverage of viral genome sequences and from other sources by simplified upstream sample preparation and downstream sequencing^23^,^24^. Furthermore, the nucleotide sequencing of the terminal ends can be conducted with ease, while the total time required for dsRNA genome sequencing shortened from months^25^ to a week or less.

## Results and Discussion

### mRCA-NGS

**Figure 1:**
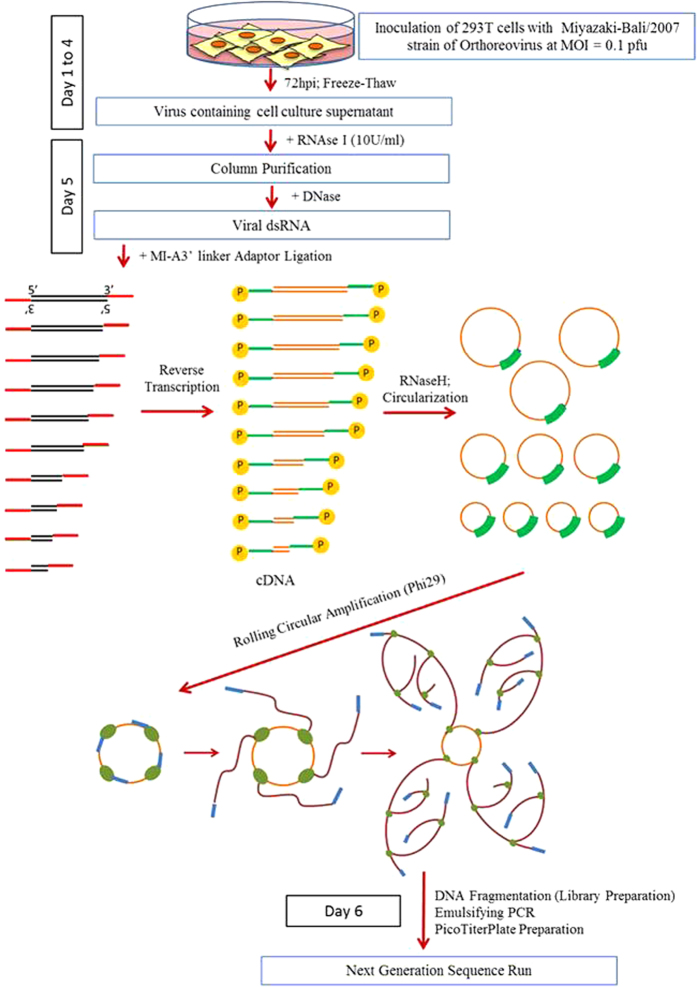
Schematic diagram for Miyazaki-Bali/2007 PRV (dsRNA) purification and novel combination method for the whole genome sequencing of dsRNA viruses; mRCA-NGS. The method involved a combination of the first step of purification of RNA with nuclease treatment, second step of adaptor ligation followed by cDNA circularization, and third step of circularized cDNA amplification using Phi29 polymerase. The use of nucleases, especially DNase treatment, was indispensable to the elimination of host genomic contamination. An MI-A3′ adaptor (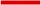) was ligated to the 3′ terminal ends of the viral dsRNA (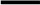) to enable easy identification of the terminals. This was then reverse transcribed into cDNA (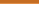) by the 5′-phosphorylated primer (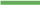), the sequence of which was complementary of MI-A3′ adaptor, followed by circularization with CircuLigase. Rolling circular amplification (RCA) was performed on the circularized cDNA with Phi29 DNA polymerase (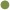) and random hexamer primers (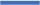) to multiple copy concatemers (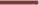). Black boxes, the number of days required for the step(s) described. MOI, pfu and hpi represent the number of viruses used for infection [multiplicity of infection (MOI)] of 293T cells expressed as plaque forming units (pfu) and the number of hours after infection [hours post infection (hpi)]-to-harvest of virus containing culture supernatant. (Figure prepared by H.S., T.Y., M. Shimojima and M. Saijo)

The sample used for NGS was prepared beginning with RNA purification from 293T cells infected with Miyazaki-Bali/2007 PRV which was cultured for 3 days, then subjected to MI-A3′ adaptor ligation, reverse transcription and cDNA circularization followed by cDNA amplification (mRCA) (Fig. 1).

**Figure 2:**
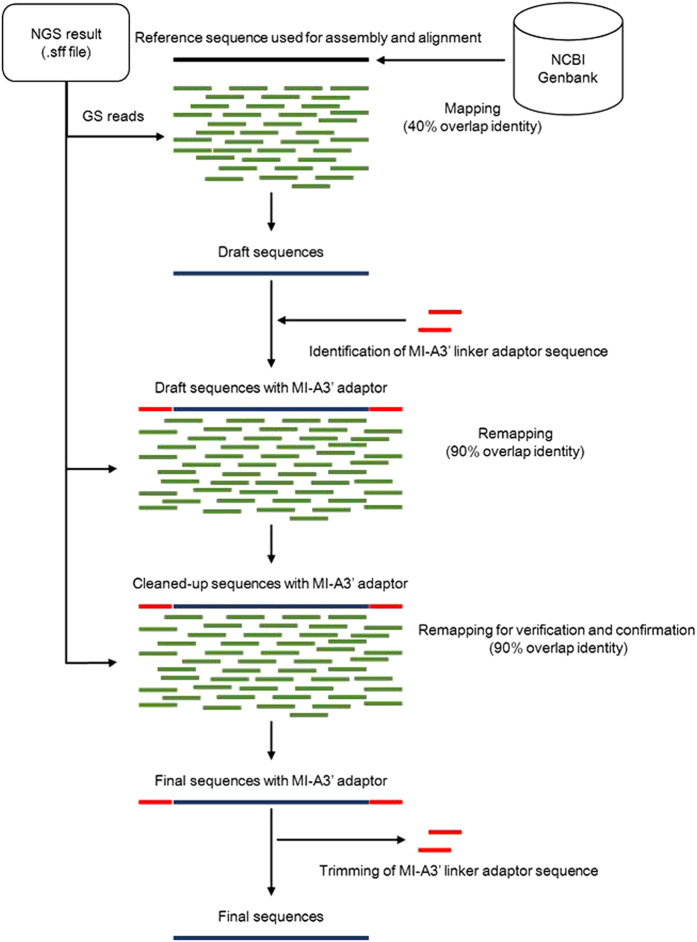
Schematic diagram for the genomic sequence assembly of PRV Miyazaki-Bali/2007 strain based on sequencing data obtained by mRCA-NGS. Raw sequence data was first assembled and aligned to the genomic segment sequence of other orthoreovirus strains (used as reference sequences) obtained through GenBank® using GS Reference Mapper software with minimal overlap identity modified from 90% to 40%. The resulting draft sequences were subsequently cleaned-up; a process now simplified by the easy identification of the MI-A3′ linker sequence. Upon sequence verification, the trimming of the MI-A3′ adaptor sequence was performed as a final step in the sequence assembly to generate the final sequences.

**Figure 3:**
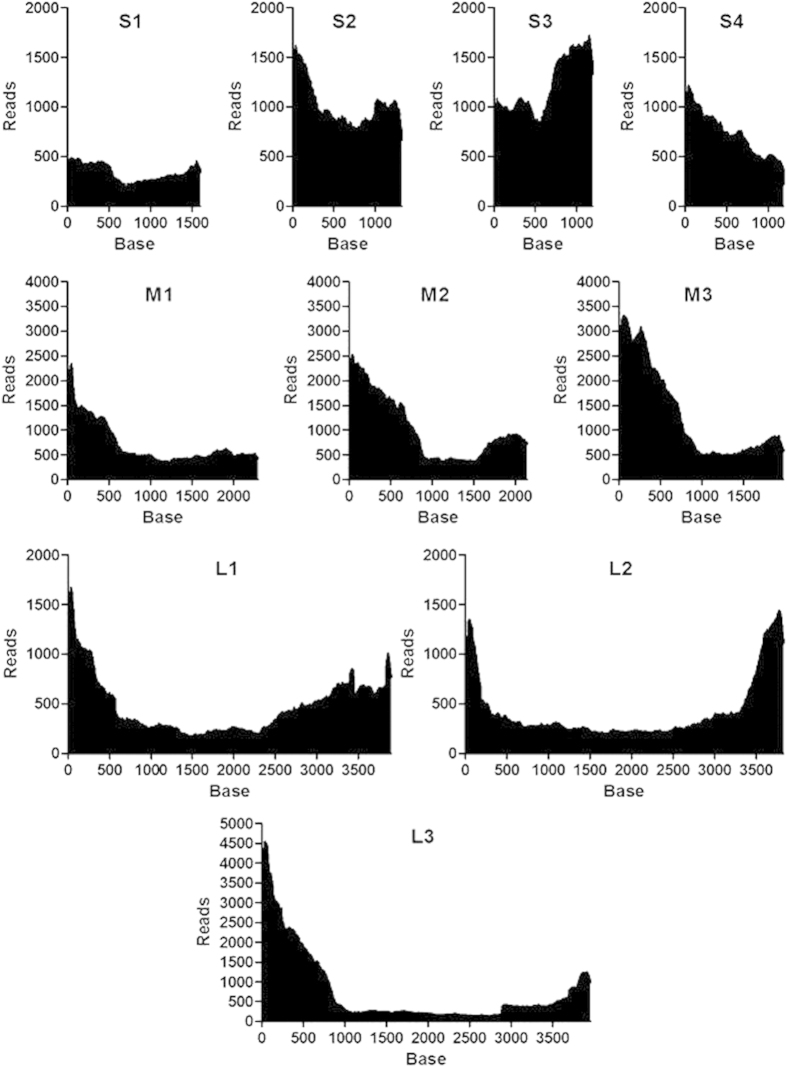
Read coverage of Miyazaki-Bali/2007 PRV strain. Read coverage shown by individual [large (L) (L1-3), medium (M) (M1-3), and small (S) (S1-4)] size-class RNA segments using data generated from mRCA-NGS. The number of reads (*y*-axis) obtained at each nucleotide base pair position (*x*-axis) is shown.

Subsequent to sequencing using GS Junior, the sequence reads were assembled and aligned to the following references: L1-3, M1-3, and S2 and 3 of Pulau PRV, or to S1 and S4 of Kampar PRV (for accession numbers see Supplementary Fig. S1). The RT primer sequence (5′-ATTGACCCGAGTTACAG-3′), complementary to the sequence of the MI-A3′ linker was added to the draft contigs that were obtained following reference-guided alignment and were re-aligned and brushed up. A schematic diagram of the assembly process of the genomic nucleotide sequence of Miyazaki-Bali/2007 PRV based on the sequence data obtained by mRCA-NGS is shown in Fig. 2. In total, 75,730 reads were obtained by this method with 66,475 reads (87.8%) aligned to the Miyazaki-Bali/2007 PRV genome. The average length of aligned reads was 248. The average read depth through the segments was 732 (minimum 136, maximum 4524). The number of reads corresponding to the coverage of the terminal regions, especially of the L-segment genomes, was higher than other portions of the segment (Fig. 3). Among the possible reasons for this is due to the cDNA extension length in the RT step using a 5′ phosphorylated RT primer. This highlights the ease by which sequences of terminal segments can be obtained using our method. The read coverage of Miyazaki-Bali/2007 PRV strain by individual segments from data generated by mRCA-NGS is shown in Fig. 3. The complete genome sequences of all segments (L1–L3, M1–M3 and S1-S4) were deposited in GenBank® with accession numbers AB908278-AB908287.

We demonstrated that the use of an MI-A3′ linker before RCA allowed for easier identification of the terminal ends in the viral dsRNA genome. Although RACE has been notably efficient in the amplification of cDNA ends^7^, the sequential combination of mRCA prior to NGS in our study proved easier to perform than standard RACE and ensured complete coverage of the whole transcript of the Miyazaki-Bali/2007 PRV strain compared to other methods of sample processing for NGS^22^. The ease and rapidity of the performance of mRCA-NGS reduced the total time required to obtain the full genome sequence of Miyazaki-Bali/2007 PRV strain [sample preparation, RNA purification and enrichment (3–4 days), mRCA (1 day or less), and NGS (1 day or less)].

In addition, the practical combination of mRCA with NGS may provide a good platform for high-throughput sequencing of similar viruses, exemplifying the potential expansion of this technology to unknown, poorly understood viruses and other viruses. As observed in this study, the reduction in the time and effort required (from one month to a total of 6–7 days) from virus supernatant preparation and extraction of structurally intact RNA to the assembly of the complete genome sequence data and the potential application to other viruses, some with greater virulence potential, cannot be excluded. Similarly, this method may also be applied in the determination of genome sequence from other non-viral sources^23^,^24^.

### Sanger dideoxy sequencing

RNA of Miyazaki-Bali/2007 PRV infected 293T cells were subjected to Sanger dideoxy sequencing. No mismatches were noted between the sequences obtained through either method, thus validating our mRCA-NGS method. Although the reads obtained by the NGS were suggestively unmatched with those obtained by first generation sequencing methods^7^, the novel combination of mRCA with NGS was shown to be accurate in the assembly of the contiguous reads, as demonstrated in our study.

### Phylogenetic analyses

The generation of complete genome sequences is of significant importance in the discovery and understanding of pathogens. Our study showed that the results obtained from mRCA-NGS revealed the full genome sequence of the Miyazaki-Bali/2007 PRV. This made possible the performance of a more in-depth molecular and phylogenetic characterization of each of its 10 segments, which highlighted evolving features that were unique among orthoreoviruses but crucial to the Miyazaki-Bali/2007 PRV strain. A phylogenetic tree was constructed based on the amino acid sequences translated from the complete genome sequences of each segment (8 structural proteins and 2 non-structural proteins, Supplementary Fig. S1) of the different orthoreovirus strains.

Together with the Melaka, Kampar, Pulau, Nelson Bay and Indonesia/2010 orthoreoviruses, Miyazaki-Bali/2007 PRV formed a cluster that was independent from avian, baboon and mammalian orthoreovirus strains for 2 out of 3 of its M-segment encoded proteins, namely the M-segment minor inner capsid protein and the M-segment non-structural protein. Similar observations were noted for all 3 of its L-segment encoding proteins: L-segment minor inner capsid protein, core spike protein and major inner capsid protein. The taxonomic classification of orthoreoviruses is based on the sequences of the S-segments creating a new group, PRV which was previously designated *Nelson Bay orthoreovirus*^4^,^5^,^6^. This classification favors the clustering of the Miyazaki-Bali/2007 strain with the Hong Kong strains, specifically for 3 out of 4 of the S-segment encoding proteins; namely the cell attachment protein (HK46886/09 and HK50842/10), major inner and major outer capsid proteins (HK23629/07, HK46886/09 and HK50842/10, and S-segment encoding non-structural protein (HK23629/07). The same classification, however, causes a divergence of the Miyazaki-Bali/2007 strain from the Australian (Nelson Bay), Malaysian strains (Melaka, Kampar, Pulau and Sikamat) and the Indonesian strain (Indonesia/2010). The phylogenetically separated clusters, based on the available S-segment encoding proteins, suggest the possible existence of three lineages which have, over time, become geographically separated: Miyazaki-Bali/2007 and Hong Kong, Australian, and Malaysian.

Interestingly, the close phylogenetic relationship between Miyazaki-Bali/2007 and the Hong Kong strains, and that the patients of both cases had traveled to Indonesia^10^,^14^,^15^, specifically to Bali Island (HK50842/10 travel history only indicates Indonesia), and its grouping as PRV together with the Malaysian strains, specifically with Pulau^19^ and Melaka^6^,^12^ PRV in which contact with bats was reported, suggests that this infection was probably acquired from an animal vector in Bali, Indonesia. Furthermore, while the clustering of Miyazaki-Bali/2007 PRV with the Hong Kong strains is seen for most of the S-segments, there is a notable separation of Miyazaki-Bali/2007 from HK23629/2007 in the S1 encoding cell attachment protein. The lack of an epidemiological link between these cases, aside from the travel histories and the recent isolation of PRV from an Indonesian bat^22^, suggests the possible occurrence of two separate spillover events from an animal vector, most probably bat to human.

The proper understanding of the evolution of viral species, as well as the contribution of viral reservoirs and hosts in the evolution of these viruses and their potential spillover into human populations can be enhanced through the use of NGS^8^. The application of NGS in combination with mRCA allows for the rapid and precise identification of the etiological agents of outbreaks due to unknown or emerging and re-emerging viral infections, especially those caused by segmented viruses and provides room for important and in-depth molecular and phylogenetic analysis to be made, as observed in our study.

In summary, mRCA-NGS was developed as a novel method for the determination and characterization of previously unknown sequence of the Miyazaki-Bali/2007 strain of PRV. This method, which can be performed rapidly, generates accurate and contiguous reads of high quality and achieves coverage of the complete genome. The performance of this new technique, allowed a comprehensive elucidation of viral evolution and phylogeny to be made.

## Methods

### Viral supernatant preparation

293T cells were infected with PRV Miyazaki-Bali/2007 at a multiplicity of infection of 0.1. The cells were cultured in Dulbecco’s Modified Eagle Medium (DMEM) supplemented with 10% fetal bovine serum and antibiotics for 72 hours. The cells were frozen and thawed 3 times to release the virus from the cells, followed by a centrifugation at 8,000 rpm for 30 minutes. Subsequently, viral titer determination in the supernatant was carried out by standard plaque assay using Vero cell monolayer.

### Sample preparation

A total of 5 ml of supernatant containing 5 × 10^6^ plaque forming unit (pfu) of the virus was used for modified rolling circular amplification with adaptor ligation – next generation sequencing (mRCA-NGS) (Fig. 1). Several steps for the purification of viral RNA were used in this study. Cellular host RNA contained in the supernatant was removed by the treatment of the supernatant with RNase I_f_ (New England BioLabs, M0243S) at a concentration of 10 U/ml, followed by the incubation at 37 °C for 1 hour. Total nucleic acid purification was performed using the High Pure Viral Nucleic Acid Kit (Roche, 11858874001) according to the manufacturer’s instruction with a final elution volume of 100 μl of RNase-free water. Subsequently, host genomic DNA was removed using the TURBO DNA-free Kit (Ambion, Life Technologies, AM1907) as per manufacturer’s instruction followed by viral dsRNA concentration using NucleoSpin RNA Clean-up XS Kit (Takara, 740948.10) with a final elution volume of 10 μl of RNase-free water.

Adaptor ligation, using the MI-A3′ linker (5′-CTGTAACTCGGGTCAATddC-3′), of the 3′ terminal ends of the viral dsRNA was performed using the DynaExpress miRNA Cloning Kit (BioDynamics Laboratory Inc., DS330) in a final reaction volume of 20 μl followed by incubation at room temperature for 2 hours. Viral dsRNA was concentrated using NucleoSpin RNA Clean-up XS Kit in a final elution volume of 11 μl of RNase-free water.

Reverse transcription was then performed utilizing the SuperScript III Reverse Transcriptase (Life Technologies, 18080-044). Briefly, the RNA was treated at 95 °C for 2 minutes after the addition of the 5′ phosphorylated RT primer (5′-ATTGACCCGAGTTACAG-3′) (complementary to the sequence of the MI-A3′ linker) and dNTP mix and immediately placed on ice. Reverse transcription was performed in a total reaction volume of 20 μl at 25 °C for 5 minutes, 50 °C for 1 hour and 70 °C for 15 minutes, respectively. The cDNA was treated with 60 U of RNase H (New England BioLabs, M0297S) at 37 °C for 20 minutes. Following purification of RNase H-treated cDNA by Monofas DNA purification kit I (GL Sciences Inc., 5010–21530), circularization of cDNA was performed using CircLigase II ssDNA Ligase (Epicentre Biotechnologies, CL9021K) in a final reaction volume of 20 μl followed by incubation at 60 °C for 1 hour and 80 °C for 10 minutes. The circularized cDNA was then precipitated by ethanol and subsequently amplified by RCA using illustra TempliPhi DNA Amplification Kit (GE Healthcare Life Sciences, 25-6400-10) as per manufacturer’s instruction. The amplified cDNA pelleted after ethanol precipitation was dissolved in a total volume of 30 μl of TE buffer followed by measurement of DNA concentration using a Thermo Scientific NanoDrop 1000 spectrophotometer.

### Whole genome sequencing by NGS

Five hundred nanograms of the cDNA was processed for sequencing using Roche’s GS FLX Titanium chemistry following Roche’s Rapid Library Preparation and emPCR Lib-L method manual. The library was sequenced on Roche’s GS Junior sequencing system as per manufacturer’s instructions. Using Roche’s GS Reference Mapper, the sequence data obtained was used to perform a reference-guided alignment using the default parameters with the exception of the minimal overlap identity modified from 90% to 40%.

### Nucleotide sequence of orthoreoviruses

The nucleotide sequences of the orthoreovirus strains used in this this study were obtained through GenBank® (http://www.ncbi.nlm.nih.gov/genbank/) and are indicated in brackets beside the viral strain names in Supplementary Figure S1. The nucleotide sequences of Miyazaki-Bali/2007 PRV were obtained from mRCA-NGS.

### Sanger dideoxy sequencing

dsRNA purified from infected 293T cells was subjected to the Sanger dideoxy sequencing. Reverse transcription was performed using SuperScript III Reverse Transcriptase according to the manufacturer’s instruction using the reverse primer sequence identical to the 3′ end of S1 to L3 of Kampar virus. The synthesized cDNA was cloned into a pUC19 Control Vector using In-Fusion® HD Cloning Kit (Takara, Z9633N). Competent *E. coli* DH5α Competent Cells (Takara, 9057) were transformed with the pUC19 Control Vector inserted with gene of interest and then purified following bacterial overnight culture. The cloned viral genome was sequenced using BigDye Terminator v3.1 Cycle Sequencing Kit (Applied Biosystems, 4337455), and Applied Biosystems ABI3130 sequencer 3130XL Genetic Analyzer. The primer sequences used were designed based on the nucleotide sequences of the Kampar PRV strain [Segment (L: Large, M: Medium and S: Small) and segment number (Genbank® accession numbers): L1 (JF342654.1), L2 (JF342655.1), L3 (JF342656.1), M1 (JF342657.1), M2 (JF342658.1), M3 (JF342659.1), S1 (EU448334.1), S2 (EU448335.1), S3 (EU448336.1) and S4 (EU448337.1)].

### Phylogenetic analyses

The orthoreovirus ORF peptide sequence data obtained in this study and those obtained through the Genbank® were phylogenetically analyzed using Multiple Sequence Comparison by Log- Expectation (MUSCLE) and the built-in program of MEGA6 (PMID: 24132122). Evolutionary distances as peptide sequences were estimated using Poisson model and phylogenetic trees were constructed using the maximum-likelihood method. The robustness of the tree was tested using 1,000 bootstrap replication.

## Additional Information

**Accession codes: GenBank®**: AB908278 - AB908287

**How to cite this article**: Singh, H. *et al*. Rapid whole genome sequencing of Miyazaki-Bali/2007 *Pteropine orthoreovirus* by modified rolling circular amplification with adaptor ligation - next generation sequencing. *Sci. Rep*. **5**, 16517; doi: 10.1038/srep16517 (2015).

## Supplementary Material

Supplementary Information
