# Supplementary material for: Rapid whole genome sequencing of Miyazaki-Bali/2007 Pteropine orthoreovirus by modified rolling circular amplification with adaptor ligation – next generation sequencing
Source: Sci Rep. 2015 Nov 12;5:16517. doi: 10.1038/srep16517 (PMC4642344; doi:10.1038/srep16517)
Supplement: Supplementary Information [file srep16517-s1.pdf]

Rapid whole genome sequencing of Miyazaki-Bali/2007 *Pteropine Orthoreovirus* by modified rolling circular amplification with adaptor ligation – next generation sequencing

Supplemental Information

Harpal Singh[1, 2, +], Tomoki Yoshikawa[2, +], Takeshi Kobayashi[3], Shuetsu Fukushi[2], Hideki Tani[2], Satoshi Taniguchi[2], Aiko Fukuma[2], Ming Yang[1], Masami Sugamata[4], Masayuki Shimojima[2] & Masayuki Saijo[2, *]

[1] Department of Intelligent Mechanical Systems, Graduate School of System Design, Tokyo Metropolitan University, Tokyo, 192-0065, Japan.

[2] Special Pathogens Laboratory, Department of Virology 1, National Institute of Infectious Diseases, Tokyo, 208-0011, Japan.

[3] Laboratory of Viral Replication, International Research Center for Infectious Diseases, Research Institute for Microbial Diseases, Osaka University, Osaka, 565-0871, Japan.

[4] Department of Hygiene and Public Health, Graduate School of Human Health Sciences, Tokyo Metropolitan University, Tokyo, 192-0397, Japan.

[*]Correspondence should be addressed to M.Saijo (email:msaijo@nih.go.jp)

[+] These authors contributed equally to this work.

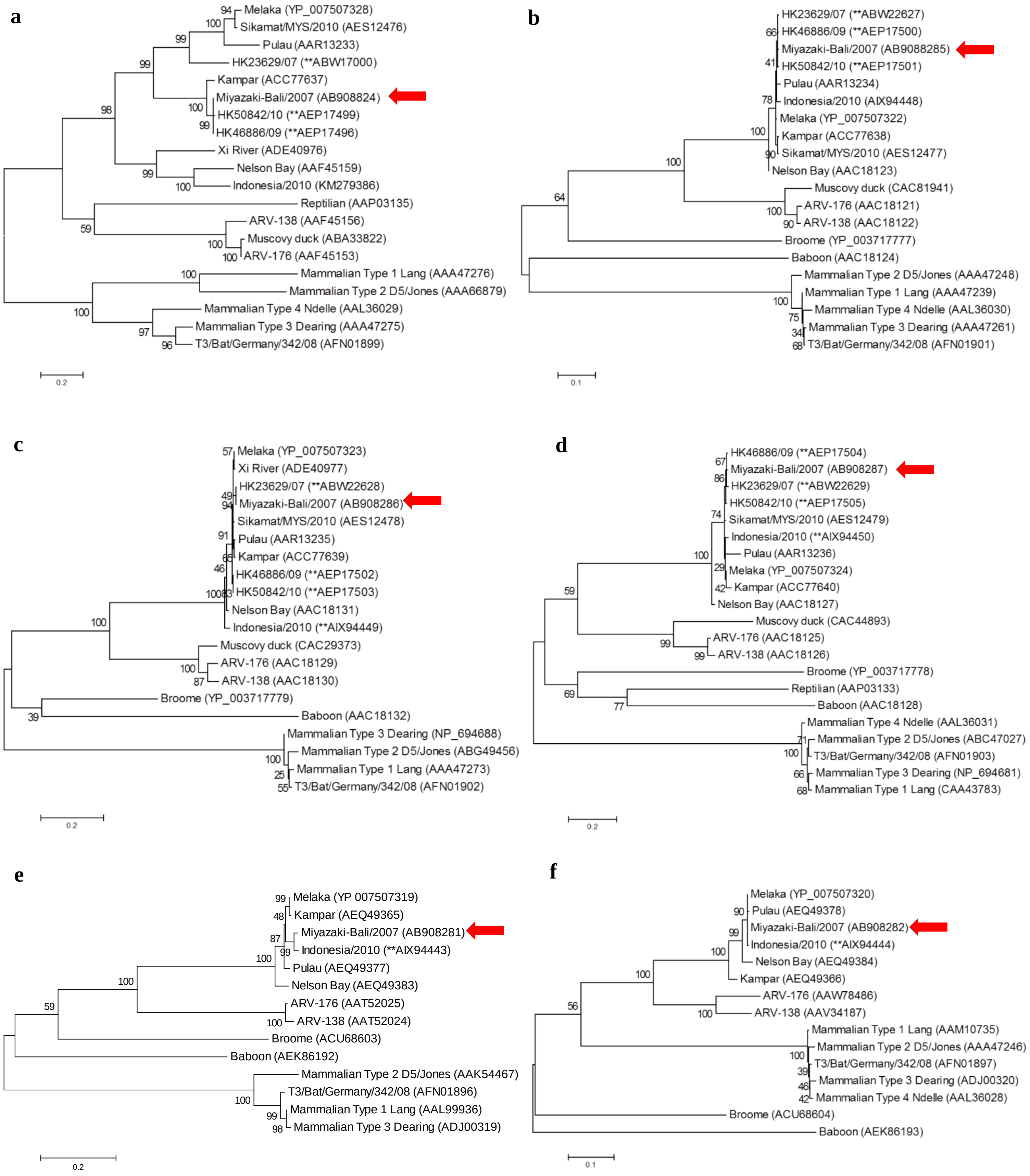
a
Melaka (YP_007507328)
Sikamat/MYS/2010 (AES12476)
Pulau (AAR13233)
HK23629/07 (**ABW17000)
Kampar (ACC77637)
Miyazaki-Bali/2007 (AB908824)
HK50842/10 (**AEP17499)
HK46886/09 (**AEP17496)
Xi River (ADE40976)
Nelson Bay (AAF45159)
Indonesia/2010 (KM279386)
Reptilian (AAP03135)
ARV-138 (AAF45156)
Muscovy duck (ABA33822)
ARV-176 (AAF45153)
Mammalian Type 1 Lang (AAA47276)
Mammalian Type 2 D5/Jones (AAA66879)
Mammalian Type 4 Ndelle (AAL36029)
Mammalian Type 3 Dearing (AAA47275)
T3/Bat/Germany/342/08 (AFN01899)
0.2
b
HK23629/07 (**ABW22627)
HK46886/09 (**AEP17500)
Miyazaki-Bali/2007 (AB9088285)
HK50842/10 (**AEP17501)
Pulau (AAR13234)
Indonesia/2010 (AIX94448)
Melaka (YP_007507322)
Kampar (ACC77638)
Sikamat/MYS/2010 (AES12477)
Nelson Bay (AAC18123)
Muscovy duck (CAC81941)
ARV-176 (AAC18121)
ARV-138 (AAC18122)
Broome (YP_003717777)
Baboon (AAC18124)
Mammalian Type 2 D5/Jones (AAA47248)
Mammalian Type 1 Lang (AAA47239)
Mammalian Type 4 Ndelle (AAL36030)
Mammalian Type 3 Dearing (AAA47261)
T3/Bat/Germany/342/08 (AFN01901)
0.1
c
Melaka (YP_007507323)
Xi River (ADE40977)
HK23629/07 (**ABW22628)
Miyazaki-Bali/2007 (AB908286)
Sikamat/MYS/2010 (AES12478)
Pulau (AAR13235)
Kampar (ACC77639)
HK46886/09 (**AEP17502)
HK50842/10 (**AEP17503)
Nelson Bay (AAC18131)
Indonesia/2010 (**AIX94449)
Muscovy duck (CAC29373)
ARV-176 (AAC18129)
ARV-138 (AAC18130)
Broome (YP_003717779)
Baboon (AAC18132)
Mammalian Type 3 Dearing (NP_694688)
Mammalian Type 2 D5/Jones (ABG49456)
Mammalian Type 1 Lang (AAA47273)
T3/Bat/Germany/342/08 (AFN01902)
0.2
d
HK46886/09 (**AEP17504)
Miyazaki-Bali/2007 (AB908287)
HK23629/07 (**ABW22629)
HK50842/10 (**AEP17505)
Sikamat/MYS/2010 (AES12479)
Indonesia/2010 (**AIX94450)
Pulau (AAR13236)
Melaka (YP_007507324)
Kampar (ACC77640)
Nelson Bay (AAC18127)
Muscovy duck (CAC44893)
ARV-176 (AAC18125)
ARV-138 (AAC18126)
Broome (YP_003717778)
Reptilian (AAP03133)
Baboon (AAC18128)
Mammalian Type 4 Ndelle (AAL36031)
Mammalian Type 2 D5/Jones (ABC47027)
T3/Bat/Germany/342/08 (AFN01903)
Mammalian Type 3 Dearing (NP_694681)
Mammalian Type 1 Lang (CAA43783)
0.2
e
Melaka (YP 007507319)
Kampar (AEQ49365)
Miyazaki-Bali/2007 (AB908281)
Indonesia/2010 (**AIX94443)
Pulau (AEQ49377)
Nelson Bay (AEQ49383)
ARV-176 (AAT52025)
ARV-138 (AAT52024)
Broome (ACU68603)
Baboon (AEK86192)
Mammalian Type 2 D5/Jones (AAK54467)
T3/Bat/Germany/342/08 (AFN01896)
Mammalian Type 1 Lang (AAL99936)
Mammalian Type 3 Dearing (ADJ00319)
0.2
f
Melaka (YP_007507320)
Pulau (AEQ49378)
Miyazaki-Bali/2007 (AB908282)
Indonesia/2010 (**AIX94444)
Nelson Bay (AEQ49384)
Kampar (AEQ49366)
ARV-176 (AAW78486)
ARV-138 (AAV34187)
Mammalian Type 1 Lang (AAM10735)
Mammalian Type 2 D5/Jones (AAA47246)
T3/Bat/Germany/342/08 (AFN01897)
Mammalian Type 3 Dearing (ADJ00320)
Mammalian Type 4 Ndelle (AAL36028)
Broome (ACU68604)
Baboon (AEK86193)
0.1

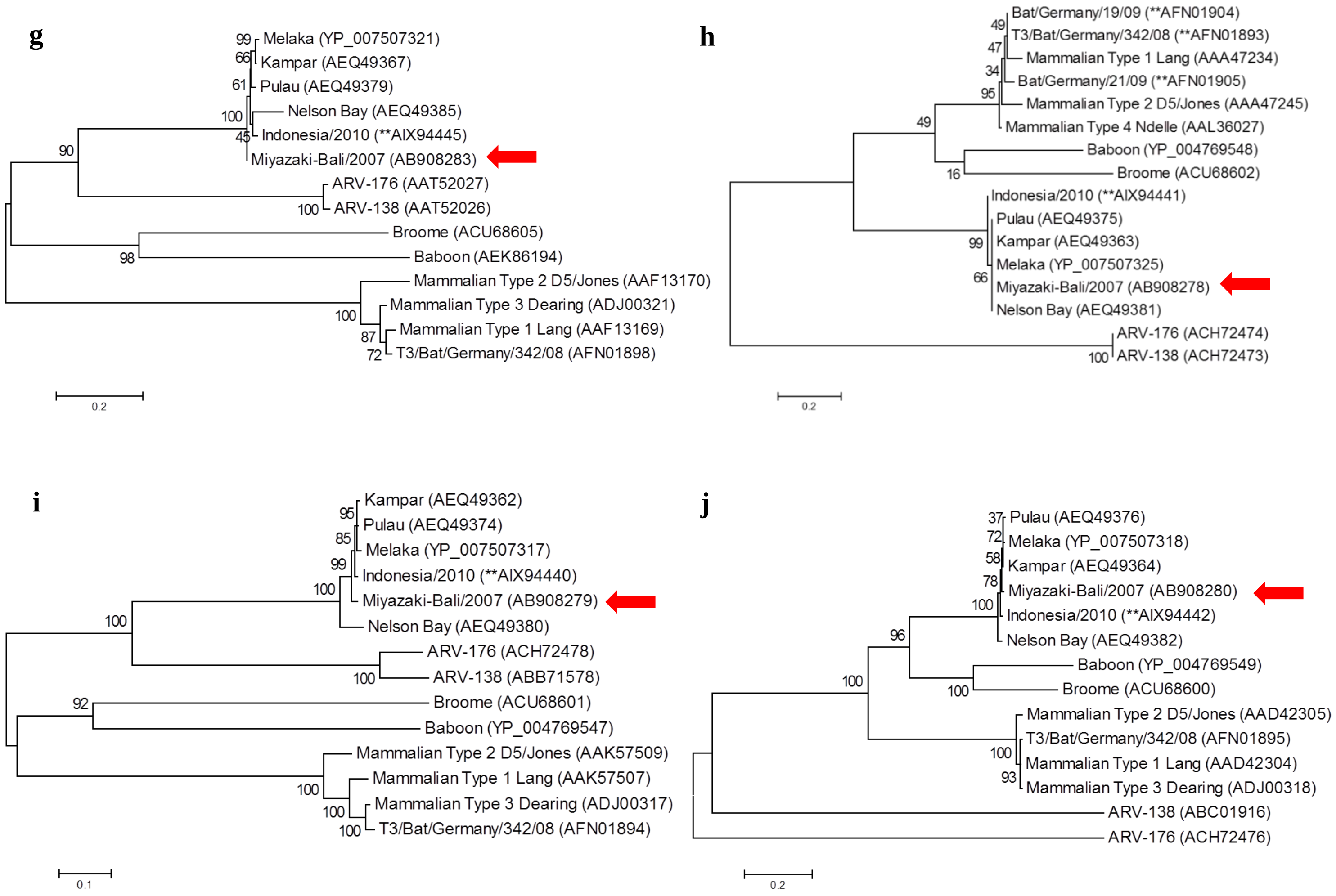
g
Melaka (YP_007507321)
Kampar (AEQ49367)
Pulau (AEQ49379)
Nelson Bay (AEQ49385)
Indonesia/2010 (**AIX94445)
Miyazaki-Bali/2007 (AB908283)
ARV-176 (AAT52027)
ARV-138 (AAT52026)
Broome (ACU68605)
Baboon (AEK86194)
Mammalian Type 2 D5/Jones (AAF13170)
Mammalian Type 3 Dearing (ADJ00321)
Mammalian Type 1 Lang (AAF13169)
T3/Bat/Germany/342/08 (AFN01898)
99
66
61
100
45
90
100
98
100
87
72
0.2
h
Bat/Germany/19/09 (**AFN01904)
T3/Bat/Germany/342/08 (**AFN01893)
Mammalian Type 1 Lang (AAA47234)
Bat/Germany/21/09 (**AFN01905)
Mammalian Type 2 D5/Jones (AAA47245)
Mammalian Type 4 Ndelle (AAL36027)
Baboon (YP_004769548)
Broome (ACU68602)
Indonesia/2010 (**AIX94441)
Pulau (AEQ49375)
Kampar (AEQ49363)
Melaka (YP_007507325)
Miyazaki-Bali/2007 (AB908278)
Nelson Bay (AEQ49381)
ARV-176 (ACH72474)
ARV-138 (ACH72473)
49
47
34
95
49
16
99
66
100
0.2
i
Kampar (AEQ49362)
Pulau (AEQ49374)
Melaka (YP_007507317)
Indonesia/2010 (**AIX94440)
Miyazaki-Bali/2007 (AB908279)
Nelson Bay (AEQ49380)
ARV-176 (ACH72478)
ARV-138 (ABB71578)
Broome (ACU68601)
Baboon (YP_004769547)
Mammalian Type 2 D5/Jones (AAK57509)
Mammalian Type 1 Lang (AAK57507)
Mammalian Type 3 Dearing (ADJ00317)
T3/Bat/Germany/342/08 (AFN01894)
95
85
99
100
100
100
92
100
100
100
0.1
j
Pulau (AEQ49376)
Melaka (YP_007507318)
Kampar (AEQ49364)
Miyazaki-Bali/2007 (AB908280)
Indonesia/2010 (**AIX94442)
Nelson Bay (AEQ49382)
Baboon (YP_004769549)
Broome (ACU68600)
Mammalian Type 2 D5/Jones (AAD42305)
T3/Bat/Germany/342/08 (AFN01895)
Mammalian Type 1 Lang (AAD42304)
Mammalian Type 3 Dearing (ADJ00318)
ARV-138 (ABC01916)
ARV-176 (ACH72476)
37
72
58
78
100
96
100
100
100
93
0.2

Supplementary Figure S1. The maximum-likelihood trees showing the phylogenetic positions of PRV Miyazaki-Bali/2007 strain (red arrow) and the others based on deduced amino acid sequence translated from the nucleotide sequence generated from mRCA-NGS. These include S-segment encoding proteins: cell attachment protein (**a**), major inner capsid protein (**b**), non-structural protein (**c**), major outer capsid protein (**d**); M-segment encoding proteins: minor inner capsid protein (**e**), major outer capsid protein (**f**), non-structural protein (**g**) and L-segment encoding proteins: minor inner capsid protein (**h**), core spike protein (**i**) and major inner capsid protein (**j**). Bootstrap values on each node are shown as the robustness of the tree. Phylogenetic trees were constructed based on: accession numbers of sequences indicated in brackets beside each viral strain and nucleotide sequence from mRCA-NGS for PRV Miyazaki-Bali/2007 strain. Accession numbers indicated beside Miyazaki-Bali/2007 are accession number deposited in GenBank®. ** = partial sequence. The values of the bootstrap percentage are shown next to the branches.
